# Integration of the Natural Language Processing of Structural Information Simplified Molecular-Input Line-Entry System Can Improve the In Vitro Prediction of Human Skin Sensitizers

**DOI:** 10.3390/toxics12020153

**Published:** 2024-02-16

**Authors:** Jae-Hee Kwon, Jihye Kim, Kyung-Min Lim, Myeong Gyu Kim

**Affiliations:** College of Pharmacy, Ewha Womans University, Seoul 03760, Republic of Korea; kkjjhh001127@naver.com (J.-H.K.); jihyekim11222@naver.com (J.K.)

**Keywords:** skin sensitizer, natural language processing, QSAR, SENS-IS, direct peptide reactivity assay (DPRA)

## Abstract

Natural language processing (NLP) technology has recently used to predict substance properties based on their Simplified Molecular-Input Line-Entry System (SMILES). We aimed to develop a model predicting human skin sensitizers by integrating text features derived from SMILES with in vitro test outcomes. The dataset on SMILES, physicochemical properties, in vitro tests (DPRA, KeratinoSens^TM^, h-CLAT, and SENS-IS assays), and human potency categories for 122 substances sourced from the Cosmetics Europe database. The ChemBERTa model was employed to analyze the SMILES of substances. The last hidden layer embedding of ChemBERTa was tested with other features. Given the modest dataset size, we trained five XGBoost models using subsets of the training data, and subsequently employed bagging to create the final model. Notably, the features computed from SMILES played a pivotal role in the model for distinguishing sensitizers and non-sensitizers. The final model demonstrated a classification accuracy of 80% and an AUC-ROC of 0.82, effectively discriminating sensitizers from non-sensitizers. Furthermore, the model exhibited an accuracy of 82% and an AUC-ROC of 0.82 in classifying strong and weak sensitizers. In summary, we demonstrated that the integration of NLP of SMILES with in vitro test results can enhance the prediction of health hazard associated with chemicals.

## 1. Introduction

Skin sensitizers are chemicals capable of inducing skin hypersensitivity [1], a condition that can progress to allergic contact dermatitis [2]. Consequently, the identification and regulation of skin sensitizers are imperative in compliance with chemicals and cosmetics regulations [3]. Traditional methods, such as the murine local lymph node assay (LLNA), have been employed for the identification of skin sensitizers [4]. LLNA determines the extent of lymph node cell proliferation induced by a test chemical, classifying the potency of skin sensitizers as strong or weak based on EC3.0 (Effective Concentration 2). EC3.0 represents the concentration of a test chemical inducing a threefold increase in lymph node cell proliferation.

While LLNA demonstrates a high hazard identification accuracy of 82.1% (46/56) for predicting human skin sensitizers, its potency prediction is comparatively less reliable, with an accuracy of approximately 59.6% (28/47) [5]. Moreover, the growing awareness of animal welfare has spurred the quest for alternative methods to LLNA. Numerous in silico and in vitro test methods have emerged for predicting skin sensitizers [6,7,8]. Among these, quantitative structure–activity relationship (QSAR) models, primarily reliant on physicochemical properties and molecular descriptors of a test chemical, have exhibited promising outcomes. Notably, the recently introduced Skin Doctor CP utilizes molecular descriptors calculated with RDKit and achieves an accuracy ranging from 75% to 89% in classifying sensitizers and non-sensitizers when compared to LLNA results [1].

Interestingly, chemicals can be represented as two-dimensional graphs, and the graph convolutional network (GCN) model can extract additional features from the graph structures [7]. Jeon et al. have pioneered the development of a graph-based ensemble machine learning model for skin sensitizers. This innovative model demonstrated an 88% accuracy (22/25) in hazard identification (sensitizers vs. non-sensitizers) using the feature set of GCN, KeratinoSens™, and h-CLAT. Furthermore, the potency prediction model for distinguishing strong sensitizers, weak sensitizers, or non-sensitizers exhibited a notable 64% accuracy (16/25) with the inclusion of GCN, DPRA, KeratinoSens™, and h-CLAT, surpassing the accuracy of LLNA at 59.6% (28/47). Recently, natural language processing (NLP) technology has garnered increasing attention. This remarkable progress is driven by models like Bidirectional Encoder Representations from Transformers (BERT) and Generative Pre-trained Transformer 4 (GPT-4), the foundational model for ChatGPT [9,10]. NLP technology enables computers to understand and generate text in ways that were previously unimaginable, making human–computer interactions more natural and effective. By applying NLP techniques to grasp and interpret chemical structures presented in Simplified Molecular-Input Line-Entry System (SMILES) notation, chemical informatics has undergone a revolutionary transformation across various domains, encompassing chemical design, property prediction, and gaining insights into chemical reactions [11]. Innovative models like ChemBERTa, MolBERT, and SMILES-BERT have emerged as powerful tools in this endeavor [12,13,14]. In this study, we aimed to develop a model predicting human skin sensitizers by integrating text features derived from SMILES using NLP with in vitro test outcomes.

## 2. Materials and Methods

### 2.1. Data Collection

The Cosmetics Europe database was used to develop a machine learning model for skin sensitization prediction. This database encompasses 6 physicochemical properties (molecular weight, octanol-water partition coefficient (LogP), water solubility (LogS), boiling point (BP), melting point (MP), vapor pressure (LogVP), bioconcentration factor (LogBCF)) and the outcomes of five in vitro tests (DPRA, KeratinoSens^TM^, h-CLAT, U-SENS^TM^, and SENS-IS) for 128 different substances [15].

A total of 1444 descriptors were further collected using PaDEL-descriptor [16]. Additionally, 22 physicochemical descriptors, previously incorporated in our skin irritation model [17], were sourced from the ChemTunes™·ToxGPS (https://mn-am.com/products/chemtunestoxgps/ (accessed on 3 January 2024)): HAccO, HAccN, HDon, HDonO, HDonN, Ro5Viol, Stereo, Complex, ComplexRing, TPSA, Dipole, Polariz, LogS, Aspheric, Eccentric, InertiaX, InertiaY, Rgyr, HoF:AM1:Cor3D:ori1, Homo:AM1:Cor3D:ori1, Lumo:AM1:Cor3D:ori1, and HomoLumoGap:AM1:Cor3D:ori1. The set of 22 physicochemical descriptors includes quantum-mechanical descriptors, which are known to better explain biological activity.

In the Cosmetics Europe database, substances were represented in the Daylight SMILES format. However, for compatibility with the ChemBERTa model used in this study, SMILES notations were collected from PubChem database (https://pubchem.ncbi.nlm.nih.gov/ (accessed on 3 January 2024)). Six substances, which are natural extracts without available SMILES notations, were excluded from this study.

The database included results of human potency categories from two previous studies [18,19]. The evidence consisted of data from human maximization tests, human repeat insult patch tests, and diagnostic patch tests. Skin sensitization in humans has been categorized into six potency categories, with categories 5 and 6 representing non-sensitizers, while categories 1 through 4 correspond to sensitizers ranging from extreme to weak [15].

### 2.2. Variables and Data Processing

In the examination of physicochemical properties, a comprehensive total of 1472 variables were taken into account, encompassing 1444 from PaDEL, 22 from ChemTunes™, and an additional 6 from the Cosmetics Europe database. To streamline the dataset, variables demonstrating a Pearson correlation coefficient of 0.75 or higher were systematically excluded, with the exception of a representative one. This refinement process resulted in a final set of 74 retained variables. Subsequently, standardized feature scaling was implemented on these variables, ensuring a mean of 0 and a variance of 1 for enhanced consistency in the analytical process.

DRPA determines the reactivity of a test substance with synthetic peptides containing cysteine (C) and lysine (K), as a means of assessing its potential to haptenize peptides in vivo [15,20]. The results were presented as data for relative C- and K-peptide depletion, along with binary interpretation according to the OECD test guidelines 442C [15]. In this study, an evaluation was conducted for both continuous variables (C- and K-peptide depletion) and binary interpretation. Ultimately, the percentages of C- and K-peptide depletion were utilized as variables, in continuous form, as they demonstrated better model performance compared to inputting the data in binary format.

KeratinoSens^TM^ assesses the activation of the Keap1-Nrf2-ARE pathway by a test substance in an adherent cell line derived from human keratinocytes stably transfected with a luciferase gene [21]. In this study, both the luciferase induction data of EC1.5, which represents the interpolated concentration inducing a 1.5-fold response compared to the vehicle control, and the binary interpretation according to the OECD test guideline 442D were used. In cases where the value of EC1.5 exceeded 2000, it was substituted with 2000 due to the difficulty in obtaining precise measurements. Finally, EC1.5 data were adopted as continuous variables, manifesting superior model performance in contrast to employing binary-formatted data.

h-CLAT assesses the ability of a substance to activate and mobilize dendritic cells in the skin by measuring the induction of the CD86 and CD54 cell surface markers [22]. The binary interpretation in accordance with the OECD test guideline 442E was used in the model development.

SENS-IS uses quality-controlled reconstituted human epidermis and predicts skin sensitization potency using the relative expression of SENS-IS and Redox genes [23]. The substances were classified into extreme, strong, moderate, weak sensitizers, and negatives. In this study, sensitizers from extreme to weak were grouped as ‘positive’ for binary classification.

The U-SENS^TM^ data were not used in the study due to their absence for 17 substances (14%). Some in vitro tests had missing values, and data with missing values were removed during the model development.

The SMILES representations of substances were analyzed using a pretrained ChemBERTa model [14]. The ChemBERTa model is built upon the RoBERTa transformer architecture with 12 attention heads and 6 layers, having been initially trained on a dataset encompassing 10 million PubChem entries [14]. The SMILES text was subjected to tokenization, with each token then converted into a unique integer by the tokenizer. The resulting sequence of token IDs, presented as a list, served as input for the ChemBERTa model. An embedding layer was employed to map each token to a 768-dimensional vector. The encoder of the model consists of 6 RoBERTa layers, each equipped with a self-attention module applied to the input, generating a 768-dimensional output vector. To introduce regularization, a dropout with a probability of 0.1 was applied. The feature utilized for machine learning was derived by calculating the average of the 768 embeddings from the last hidden layer. This feature underwent standardized feature scaling as a further step in the analytical process.

The dataset underwent partitioning into training and testing sets at an 8:2 ratio, comprising 97 compounds in the training set and 25 compounds in the testing set. To address the imbalance observed in the human potency category within the data, a proportional adjustment was made to ensure the equal representation of category proportions between the training and testing sets.

### 2.3. Model Structure

**Figure 1:**
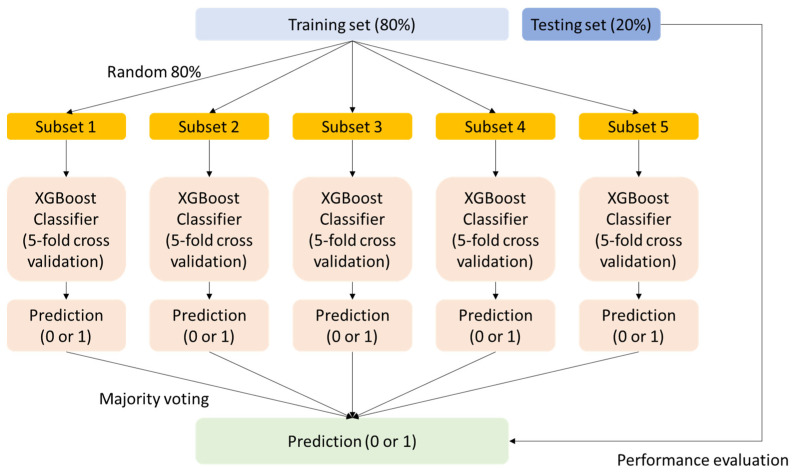
Framework of the model.

Figure 1 illustrates the model framework. Given the relatively modest size of the dataset, this study utilized the bagging-XGBoost algorithm [24]. Through random sampling with replacement, five subsets were generated from the training set, with each subset encompassing 80% of the data.

The training process involved employing the Extreme Gradient Boosting (XGBoost) model on each of these subsets. During the XGBoost training phase, the optimization of hyperparameters in Table 1 was executed using 5-fold cross-validation and grid search. Given the imbalanced nature of the data, each XGBoost model underwent training with the objective of maximizing balanced accuracy. Furthermore, the introduction of the scale_pos_weight hyperparameter aimed to tackle data imbalance. Evaluation occurred under two conditions: without any weighting applied (scale_pos_weight = 1) and with scale_pos_weight (adjusted for class ratios) set to either 0.5 or 2.0.

The XGBoost models, trained on 5 subsets, were amalgamated using a majority voting approach. This involves aggregating the predictions from individual models and determining the final prediction by selecting the class that receives the majority of votes. Incorporating this bagging method aids in mitigating output variance and augmenting the algorithm’s generalization ability.

### 2.4. Modeling Strategy

**Figure 2:**
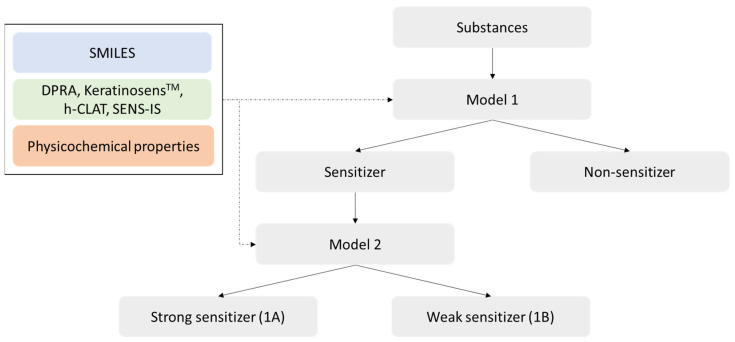
Two-stage classification modeling.

In this study, substances were classified into two stages (Figure 2). First, substances were categorized as sensitizer (potency categories 1 through 4) or non-sensitizer (potency categories 5 and 6). Subsequently, sensitizers were further classified into strong (1A; potency categories 1 and 2) or weak sensitizer (1B; potency categories 3 and 4).

The selection of variables for inclusion in the model was based on the average feature importance derived from five XGBoost models. Feature importance indicates the contribution of each feature to the model by measuring the reduction in loss when a specific feature is utilized for splitting. The ultimate model was chosen by comparing the balanced accuracy of models using the top 10 and top 15 features, as determined by feature importance.

Accuracy, balanced accuracy, AUC-ROC (area under the receiver operating characteristic curve), sensitivity, specificity, and F1 score were calculated from predicted values and actual values in the testing dataset using the following formulas [25]:

Accuracy = (TP + TN)/(TP + TN + FP + FN),(1)

Balanced accuracy = (Sensitivity + Specificity)/2,(2)

Sensitivity = TP/(TP + FN),(3)

Specificity = TN/(FP + TN),(4)

F1 score = 2 × TP/(2 × TP + FP + FN).(5)

Here, TP and TN represent true positives and negatives; FP and FN represent false positives and negatives.

The machine learning analysis was conducted in the Google Colab environment (https://colab.research.google.com/ (accessed on 3 January 2024)).

### 2.5. SHAP (SHapley Additive exPlanations)

SHAP is an explainable AI method that employs a game-theoretic approach to elucidate the outcomes of machine learning [26]. SHAP values delineate the contribution of each feature to the model’s prediction [26]. Positive SHAP values denote contributions that elevate the model’s prediction, whereas negative values indicate contributions that lower the prediction.

To calculate SHAP values, the testing set was input into a subset-trained XGBoost model. Subsequently, the SHAP values obtained from five models were averaged and visualized. The SHAP values were computed using the SHAP package within the Google Colab environment.

## 3. Results

### 3.1. Modeling Process

Figure S1 depicts the average feature importance of a model designed to discriminate sensitizers from non-sensitizers. Notably, ‘K-peptide’ and ‘BCUTc-1l’ share the 15th position, possessing identical average feature importance values. Therefore, the variables for the top 16 include C-peptide, EC1.5, SIC5, SENS-IS_cat, h-CLAT, roberta_embedding_mean, MDEC-11, VE1_Dt, Homo:AM1:Cor3D:ori1, MDEC-12, MLFER_S, nO, HomoLumoGap:AM1:Cor3D:ori1, ALogp2, K-peptide, and BCUTc-1l.

Table 2 displays the balanced accuracy of the model considering all features, the top 10 features, and the top 16 features. The model utilizing 16 features outperformed the 10-feature model, and while not surpassing the model with all features, it demonstrated an acceptable balanced accuracy. Initially, four types of in vitro tests were included. To streamline the testing process, the least important in vitro test, h-CLAT, was substituted with the most crucial feature among those not initially included in the model, BCUTp-1l. As both models exhibited identical balanced accuracy at 0.8199, the model incorporating BCUTp-1l was chosen in place of h-CLAT. Refer to Table S1 for the meanings of each feature and Table S2 for the distribution of each feature.

Figure S2 illustrates the average feature importance of a model designed to differentiate between strong and weak sensitizers. The top 15 variables, in descending order of importance, include C-peptide, C1SP3, MDEC-22, EC1.5, SIC0, RotBFrac, VE1_Dt, TSRW, BP, K-peptide, LogP, Dipole, LogS, ALogp2, and C3SP2.

Table 2 presents the balanced accuracy of the model incorporating all features, the top 10 features, and the top 15 features. The model incorporating 10 features achieved the highest balanced accuracy, leading to its selection as the final model. Notably, the six features excluding EC1.5, C-peptide, K-peptide, and VE1_Dt were not utilized in classifying sensitizers and non-sensitizers. For detailed information on each feature, refer to Table S1, and find the distribution of each feature in Table S3.

### 3.2. Model Performance

**Figure 3:**
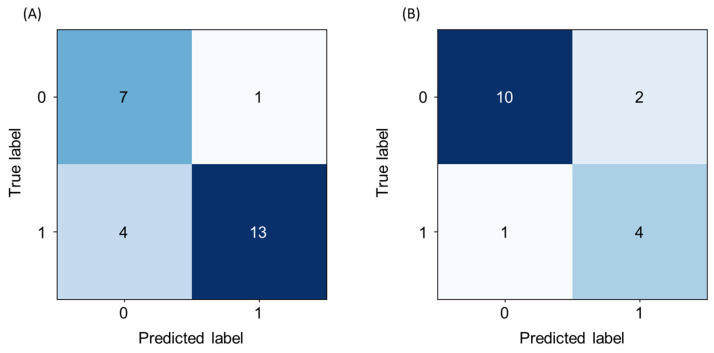
Confusion matrix of skin sensitization prediction. (**A**) Sensitizer (class 1) vs. non-sensitizer (class 0), and (**B**) strong sensitizer (class 1) vs. weak sensitizer (class 0).

Figure 3 and Table 3 illustrate the confusion matrix and predictive performance for the two final models, respectively. The first model designed to distinguish sensitizers from non-sensitizers demonstrated commendable performance for the test dataset composed of 25 substances (8 non-sensitizers and 17 sensitizers), yielding an accuracy of 0.8 and an AUC-ROC of 0.82. Likewise, the second model designed to classify strong and weak sensitizers achieved an accuracy of 0.82 and an AUC-ROC of 0.82 for 17 sensitizers (12 weak sensitizers and 5 strong sensitizers) of the test dataset.

The overall accuracy of the potency prediction of sensitizers using the combined two-stage model was 72% (18/25; Table 4). Of the strong sensitizers, 20% (1/5) were underpredicted as weak sensitizers, while 12.5% (1/8) of non-sensitizers were overpredicted. Of the weak sensitizers, 33.3% (4/12) were underpredicted and 8.3% (1/8) were overpredicted.

### 3.3. SHAP Analysis

**Figure 4:**
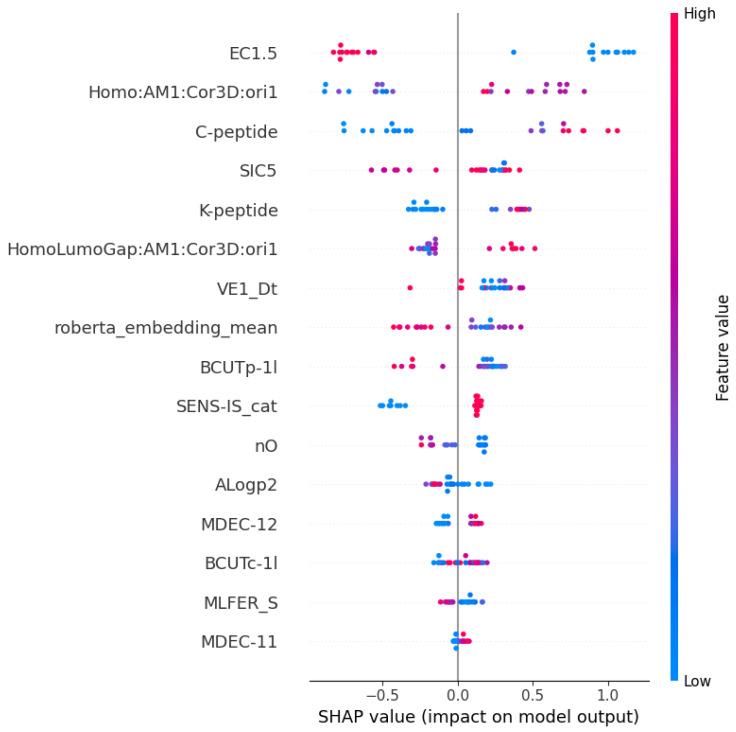
SHAP summary plot for a classifier distinguishing between sensitizers and non-sensitizers.

Figure 4 and Figure S3 illustrate the SHAP summary plot for the classification of sensitizers and non-sensitizers. On the *y*-axis, the feature list is arranged in descending order of mean SHAP values, with each feature’s color corresponding to its value—red indicating higher values and blue indicating lower values. The *x*-axis depicts the SHAP values, providing insight into the magnitude and direction of influence each feature has on the model’s output.

EC1.5 exerted the most pronounced impact on the classification between sensitizers and non-sensitizers, with smaller values indicative of categorization as a sensitizer. In contrast, the percentage of C- and K-peptide depletion and SENS-IS_cat had less influence compared to EC1.5, yet higher values of these features were associated with classification as a sensitizer.

Among the physicochemical properties, Homo:AM1:Cor3D:ori1 exhibited the highest influence, and values surpassing the average suggested a higher likelihood of being a sensitizer. Additionally, the mean of last hidden layer embeddings obtained from the ChemBERTa model ranked as the seventh most influential feature for sensitization prediction. A lower value of this feature indicated a higher likelihood of being classified as a sensitizer.

**Figure 5:**
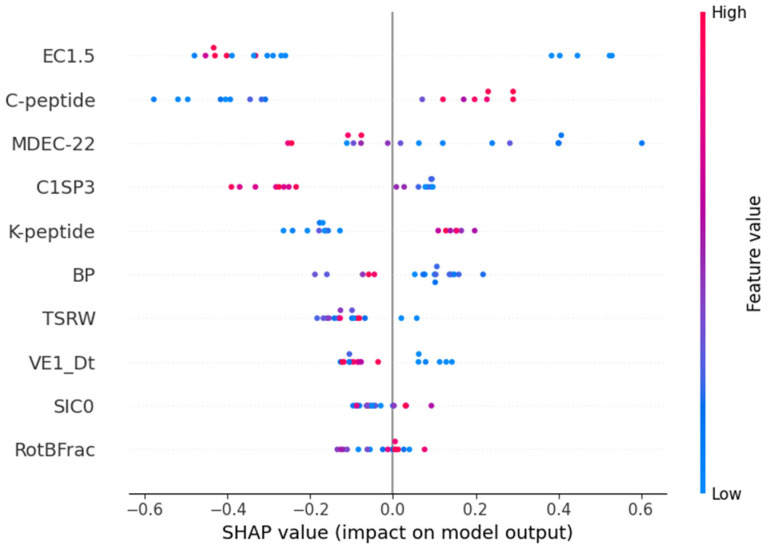
SHAP summary plot for a classifier distinguishing between strong and weak sensitizers.

Figure 5 and Figure S4 demonstrate the SHAP summary plot for the classification of strong and weak sensitizers. In this distinction, EC1.5 wielded the most substantial impact, with lower values signifying a higher likelihood of being a strong sensitizer. Conversely, higher values of C-peptide and K-peptide were associated with a greater likelihood of being a strong sensitizer. Among physicochemical properties, MDEC-22 emerged as a highly influential feature, where lower values indicated a higher likelihood of being classified as a strong sensitizer.

Figures S5 and S6 presents the average SHAP values for all substances within the testing set across five models. While the final predictions were derived through majority voting from the predictions of five XGBoost models, exceptions may exist where the sum of average SHAP values differs from the final prediction (e.g., citronellol). Despite this, it remains feasible to examine the influence of features on the classification of each substance as a sensitizer or non-sensitizer. For instance, in the case of the sensitizer penicillin G, results based on EC1.5 and SENS-IS suggest proximity to a non-sensitizer. However, employing features such as Homo:AM1:Cor3D:ori1, HomoLumoGap:AM1:Cor3D:ori1, and embedding values facilitated an accurate classification as a sensitizer.

## 4. Discussion

Here, we successfully constructed a prediction model for human skin sensitizers utilizing the SMILES data analyzed through natural language processing (NLP) techniques. Consequently, this model demonstrated outstanding predictive capabilities, achieving an accuracy of 80% (20/25) in classifying sensitizers versus non-sensitizers and distinguishing strong sensitizers from weak ones with an accuracy of 82% (14/17). The overall accuracy for potency prediction reached 72% (18/25).

Transformer models exhibit a high degree of adaptability to transfer learning, a process where a pre-trained model on one task or dataset can be fine-tuned on a different, often smaller, dataset for a specific task [27]. In a previous study, ChemBERTa demonstrated its ability to identify toxic chemicals from the ClinTox dataset and p53 stress-response pathway activators from the Tox21 dataset, achieving AUC-ROC values of 0.733 and 0.728, respectively [14]. The substantial number of data points in these datasets, 1478 from ClinTox and 7831 from Tox21, proved conducive to effective fine-tuning and alignment for diverse classification tasks. In contrast, the limited dataset comprising 122 substances from the Cosmetics Europe database in this study (with 97 utilized in the training process) proved insufficient for the accurate classification of skin sensitizers based solely on SMILES. To overcome this limitation, in vitro test results and other physicochemical properties were integrated to enhance model performance. In consolidating the SMILES analysis results for feature integration, we adopted the approach of using the average value of embeddings from the last hidden layer. This method effectively captures the representative features of the chemical structures encoded by the model, contributing to improved performance in downstream analysis.

A single in vitro test can identify human skin sensitizers with an accuracy ranging from 73.4% to 78.6% [15]. SENS-IS showed a higher accuracy of 78.6% in identifying human sensitizers compared to other in vitro tests [15]. Although SENS-IS has not been fully approved yet by OECD [28], our study demonstrated the significance of SENS-IS as an important variable in machine learning for predicting human skin sensitizers. In a previous study, DPRA appeared to be the most predictive skin sensitization test, surpassing KeratinoSens™, h-CLAT, and LLNA, with a balanced accuracy as high as 79%, sensitivity and positive predictive value above 82%, and specificity and negative predictive value above 70% [29].

To address the inherent limitations of a single in vitro test, several studies have adopted a holistic approach by incorporating multiple in vitro tests, physicochemical properties, and structural information. Zhang et al. achieved an 81% accuracy in their model, employing support vector methods with physicochemical properties, DPRA, h-CLAT, and KeratinoSens™ assay data [8]. Jeon et al. developed a graph-based ensemble machine that exhibited an 88% accuracy for hazard identification and a 64% accuracy for potency prediction, utilizing multiple in vitro tests and the structural graph of substances [7]. The current OECD Guideline 497 Integrated Testing Strategy version 2 (ITSv2), employing DPRA, h-CLAT, and the OECD Toolbox, demonstrated an accuracy of 87% (54/62) for hazard identification and 70% (40/57) for potency prediction [5]. Our model’s performance in hazard identification was comparable to these established models, achieving an accuracy of 80% and a balanced accuracy of 82%. Furthermore, our model showcased an improved accuracy of 72% (18/28) for potency prediction when contrasted with the current OECD Guideline 497 ITSv2. Undoubtedly, conducting a direct comparison poses challenges given the variations in the composition of testing sets across different studies. Nevertheless, the integration of SMILES information with in vitro tests and physicochemical properties enhances the overall performance of the model.

Typically, the dataset utilized for model development defines the applicability domain [30]. The 122 chemicals employed in developing and testing our model exhibit molecular weights (MW) ranging from 30.03 to 604.82 (with 95% below 430.5), LogP values ranging from −8.28 to 8.49 (with 95% below 6.2), and water solubility (LogS) ranging from −2.3 to 1.2 (with 95% below 1.2). Chemicals falling outside these ranges may be predicted with a higher likelihood of errors in our model. In the classification of sensitizers and non-sensitizers, 80% (4/5) of the substances incorrectly classified belonged to the category of false negatives. Unlike substances in the training set or those accurately classified in the testing set, the majority of these substances had an EC1.5 of 2000. As EC1.5 is the most influential feature in classifying sensitizers, a value of 2000 could potentially result in the misclassification of substances as false negatives. Consequently, caution is advised in interpreting substances with EC1.5 values close to 2000 when applying this model.

This study has some limitations. First, the limited amount of human data for skin sensitizers posed a constraint on the fine-tuning of ChemBERTa. Secondly, this model cannot be applied to substances that do not have PubChem standardized SMILES representations. Third, using the average embedding of SMILES as a feature means that we cannot identify the influence of each token, making it impossible to determine which structural elements are involved in sensitization. Lastly, for substances beyond the range of the training set, the predictive performance is diminished. In particular, substances with an EC1.5 of 2000 were found to have a high likelihood of being classified as false negatives.

## 5. Conclusions

The integration of the NLP of SMILES with in vitro test results could make a prediction model with an enhanced performance to predict human skin sensitizers. While the outcomes of in vitro tests exerted a significant influence on classification, the information encoded in SMILES also played a role in differentiating between sensitizers and non-sensitizers. Nevertheless, additional research is imperative to acquire a larger dataset, thereby refining the model and enhancing the overall performance of the model.

## Figures and Tables

**Table 1:** List of hyperparameter values.

Hyperparameters	Sensitizer vs. Non-Sensitizer	Strong vs. Weak Sensitizer
learning_rate	0.01, 0.1, 0.2	
n-estimators	50, 100, 200, 300, 500	
max_depth	3, 5, 7, 9	
subsample	0.6, 0.8, 1.0	
scale_pos_weight	0.5, 1.0	1.0, 2.0

**Table 2:** Balanced accuracy of models.

Model	All Features	Top 15 Features	Top 10 Features
Sensitizers vs. non-sensitizers	0.8493	0.8199 *	0.7868
Strong vs. weak sensitizers	0.7000	0.7167	0.8167

* This model includes 16 features.

**Table 3:** Performance of the final models.

Final Model	Accuracy	AUC-ROC	Sensitivity	Specificity	F1
Sensitizers vs. non-sensitizers	0.8000	0.8199	0.7647	0.8750	0.8387
Strong vs. weak sensitizers	0.8235	0.8167	0.8000	0.8333	0.7273

**Table 4:** Potency categorization performance of the final model.

	Human		
Predicted	NC	1B	1A
NC	7	4	0
1B	1	7	1
1A	0	1	4
72% correct classification overall			
	NC (N = 8)	1B (N = 12)	1A (N = 5)
Correct classification (%)	87.5%	58.3%	80%
Underpredicted (%)	NA	33.3% (NC)	20% (1B)
Overpredicted (%)	12.5% (1B)	8.3% (1A)	NA

NA, not applicable; NC, non-sensitizer; 1A, strong sensitizer; 1B, weak sensitizer.

## Data Availability

The data presented in this study are available in Supplementary materials.

## Supplementary Materials

The following supporting information can be downloaded at: https://www.mdpi.com/article/10.3390/toxics12020153/s1, Table S1: Description of the features; Table S2. Distribution of features included in the model (sensitizers vs. non-sensitizers); Table S3. Distribution of features included in the model (strong vs. weak sensitizers); Figure S1: The feature importance of a classifier distinguishing between sensitizers and non-sensitizers; Figure S2: The feature importance of a classifier distinguishing between strong and weak sensitizers; Figure S3: The average SHAP values for a classifier distinguishing between sensitizers and non-sensitizers; Figure S4: The average SHAP values for a classifier distinguishing between strong and weak sensitizers; Figure S5. SHAP force plot for test substances and predicted results (sensitizers vs. non-sensitizers); Figure S6. SHAP force plot for test substances and predicted results (strong vs. weak sensitizers).

## References

[1] Wilm A., Norinder U., Agea M.I., de Bruyn Kops C., Stork C., Kuhnl J., Kirchmair J. (2021). Skin Doctor CP: Conformal Prediction of the Skin Sensitization Potential of Small Organic Molecules. Chem. Res. Toxicol..

[2] Park H., Hwang J.H., Han J.S., Lee B.S., Kim Y.B., Joo K.M., Choi M.S., Cho S.A., Kim B.H., Lim K.M. (2018). Skin irritation and sensitization potential of oxidative hair dye substances evaluated with in vitro, in chemico and in silico test methods. Food Chem. Toxicol..

[3] Bialas I., Zelent-Kraciuk S., Jurowski K. (2023). The Skin Sensitisation of Cosmetic Ingredients: Review of Actual Regulatory Status. Toxics.

[4] Ha S., Ahn I.Y., Kim D.E., Lee J.K., Sohn S., Jung M.S., Heo Y., Omori T., Bae S., Lim K.M. (2017). Evaluation of radioisotopic and non-radioisotopic versions of local lymph node assays for subcategorization of skin sensitizers compliant to UN GHS rev 4. Regul. Toxicol. Pharmacol..

[5] OECD (2021). Guideline No. 497: Defined Approaches on Skin Sensitisation.

[6] Ambe K., Suzuki M., Ashikaga T., Tohkin M. (2021). Development of quantitative model of a local lymph node assay for evaluating skin sensitization potency applying machine learning CatBoost. Regul. Toxicol. Pharmacol..

[7] Jeon B., Lim M.H., Choi T.H., Kang B.C., Kim S. (2022). A development of a graph-based ensemble machine learning model for skin sensitization hazard and potency assessment. J. Appl. Toxicol..

[8] Zang Q., Paris M., Lehmann D.M., Bell S., Kleinstreuer N., Allen D., Matheson J., Jacobs A., Casey W., Strickland J. (2017). Prediction of skin sensitization potency using machine learning approaches. J. Appl. Toxicol..

[9] Devlin J., Chnag M.W., Lee K., Toutanova K. (2018). BERT: Pre-training of Deep Bidirectional Transformers for Language Understanding. arXiv.

[10] Open AI (2023). GPT-4 Technical Report. arXiv.

[11] Ucak U.V., Ashyrmamatov I., Lee J. (2023). Improving the quality of chemical language model outcomes with atom-in-SMILES tokenization. J. Cheminform..

[12] Fabian B., Edlich T., Gaspar H., Segler M., Meyers J., Fiscato M., Ahmed M. (2020). Molecular representation learning with language models and domain-relevant auxiliary tasks. arXiv.

[13] Wang S., Guo Y., Wang Y., Sun H., Huang J. Smiles-bert: Large scale unsupervised pre-training for molecular property prediction. Proceedings of the 10th ACM International Conference on Bioinformatics, Computational Biology and Health Informatics.

[14] Chithrananda S., Grand G., Ramsundar B. (2020). ChemBERTa: Large-scale self-supervised pretraining for molecular property prediction. arXiv.

[15] Hoffmann S., Kleinstreuer N., Alépée N., Allen D., Api A.M., Ashikaga T., Clouet E., Cluzel M., Desprez B., Gellatly N. (2018). Non-animal methods to predict skin sensitization (I): The Cosmetics Europe database. Crit. Rev. Toxicol..

[16] Yap C.W. (2011). PaDEL-descriptor: An open source software to calculate molecular descriptors and fingerprints. J. Comput. Chem..

[17] Kang Y., Kim M.G., Lim K.M. (2023). Machine-learning based prediction models for assessing skin irritation and corrosion potential of liquid chemicals using physicochemical properties by XGBoost. Toxicol. Res..

[18] Api A.M., Parakhia R., O’Brien D., Basketter D.A. (2017). Fragrances Categorized According to Relative Human Skin Sensitization Potency. Dermatitis.

[19] Basketter D.A., Alepee N., Ashikaga T., Barroso J., Gilmour N., Goebel C., Hibatallah J., Hoffmann S., Kern P., Martinozzi-Teissier S. (2014). Categorization of chemicals according to their relative human skin sensitizing potency. Dermatitis.

[20] Gerberick G.F., Vassallo J.D., Bailey R.E., Chaney J.G., Morrall S.W., Lepoittevin J.P. (2004). Development of a peptide reactivity assay for screening contact allergens. Toxicol. Sci..

[21] Emter R., Ellis G., Natsch A. (2010). Performance of a novel keratinocyte-based reporter cell line to screen skin sensitizers in vitro. Toxicol. Appl. Pharmacol..

[22] Ashikaga T., Yoshida Y., Hirota M., Yoneyama K., Itagaki H., Sakaguchi H., Miyazawa M., Ito Y., Suzuki H., Toyoda H. (2006). Development of an in vitro skin sensitization test using human cell lines: The human Cell Line Activation Test (h-CLAT). I. Optimization of the h-CLAT protocol. Toxicol. In Vitro.

[23] Cottrez F., Boitel E., Ourlin J.C., Peiffer J.L., Fabre I., Henaoui I.S., Mari B., Vallauri A., Paquet A., Barbry P. (2016). SENS-IS, a 3D reconstituted epidermis based model for quantifying chemical sensitization potency: Reproducibility and predictivity results from an inter-laboratory study. Toxicol. In Vitro.

[24] Deng X., Ye A., Zhong J., Xu D., Yang W., Song Z., Zhang Z., Guo J., Wang T., Tian Y. (2022). Bagging–XGBoost algorithm based extreme weather identification and short-term load forecasting model. Energy Rep..

[25] Kim M.G., Kim J., Kim S.C., Jeong J. (2020). Twitter Analysis of the Nonmedical Use and Side Effects of Methylphenidate: Machine Learning Study. J. Med. Internet Res..

[26] Lundberg S.M., Lee S.I. A Unified Approach to Interpreting Model Predictions. Proceedings of the 31st Conference on Neural Information Processing Systems.

[27] Kim M.G., Kim M., Kim J.H., Kim K. (2022). Fine-Tuning BERT Models to Classify Misinformation on Garlic and COVID-19 on Twitter. Int. J. Environ. Res. Public Health.

[28] Ta G.H., Weng C.F., Leong M.K. (2021). In silico Prediction of Skin Sensitization: Quo vadis?. Front. Pharmacol..

[29] Alves V.M., Capuzzi S.J., Braga R.C., Borba J.V.B., Silva A.C., Luechtefeld T., Hartung T., Andrade C.H., Muratov E.N., Tropsha A. (2018). A Perspective and a New Integrated Computational Strategy for Skin Sensitization Assessment. ACS Sustain. Chem. Eng..

[30] Weaver S., Gleeson M.P. (2008). The importance of the domain of applicability in QSAR modeling. J. Mol. Graph. Model..

