# Supplementary material for: Integration of the Natural Language Processing of Structural Information Simplified Molecular-Input Line-Entry System Can Improve the In Vitro Prediction of Human Skin Sensitizers
Source: Toxics. 2024 Feb 16;12(2):153. doi: 10.3390/toxics12020153 (PMC10892072; doi:10.3390/toxics12020153)

Table S1. Description of the features.

Feature	Description
**Sensitizer vs. non-sensitizer**	
C-peptide	Peptide reactivity of a substance towards model synthetic heptapeptides that contain cysteine
EC1.5	Interpolated concentration inducing a 1.5-fold response compared to the vehicle control
SIC5	Structural information content index (neighborhood symmetry of 5-order)
SENS-IS_cat	Skin sensitization potency using the relative expression of the SENS-IS and Redox genes
roberta_embedding_mean	Average of the 768 embeddings from the last hidden layer of ChemBERTa
MDEC-11	Molecular distance edge between all primary carbons
VE1_Dt	Coefficient sum of the last eigenvector from detour matrix
Homo:AM1:Cor3D:ori1	Energy of the highest-occupied molecular orbital using the AM1 Hamiltonian
MDEC-12	Molecular distance edge between all primary and secondary carbons
MLFER_S	Combined dipolarity/polarizability
nO	Number of oxygen atoms
HomoLumoGap:AM1:Cor3D:ori1	Energy difference of the highest occupied and the lowest unoccupied molecular orbital using the AM1s Hamiltonian
ALogp2	Square of Ghose-Crippen Log octanol/water partition coefficient (Kow)
K-peptide	Peptide reactivity of a substance towards model synthetic heptapeptides that contain lysine
BCUTc-1l	nhigh lowest partial charge weighted BCUTS
BCUTp-1l	nhigh lowest polarizability weighted BCUTS
**Strong vs. weak sensitizer**	
C-peptide	Same as above
C1SP3	Singly bound carbon bound to one other carbon
MDEC-22	Molecular distance edge between all secondary carbons
EC1.5	Same as above
SIC0	Structural information content index (neighborhood symmetry of 0-order)
RotBFrac	Fraction of rotatable bonds, excluding terminal bonds
VE1_Dt	Same as above
TSRW	Total self-return walk count (up to order 10) (ln(1+x))
BP	Boiling point
K-peptide	Same as above

Table S2. Distribution of features included in the model (sensitizer vs. non-sensitizer)

Feature†	Training set	Testing set (Correct prediction for sensitizer and non-sensitizer)	Testing set (Incorrect prediction for sensitizer and non-sensitizer)
	N = 97	N = 20	N = 5
EC1.5	115.5 (0.5, 2000) [17.3, 2000]	109.8 (1.8, 2000) [15.7, 2000]	2000 (45.4, 2000) [2000, 2000]
Homo:AM1:Cor3D:ori1	-9.56 (-12.5, 0) [-10.2, -9.07]	-9.74 (-11.3, -7.49) [-10.2, -9.25]	-9.48 (-10.7, -9.23) [-9.59, -9.31]
C-peptide	11.38 (0, 100) [1, 67.3]	18.7 (0, 100) [5.3, 80.2]	3.2 (0, 52.3) [1.6, 5.4]
SIC5	0.79 (0, 1) [0.67, 0.86]	0.82 (0, 0.92) [0.66, 0.87]	0.81 (0.79, 0.94) [0.80, 0.92]
K-peptide	3.24 (0, 100) [0, 14.6]	3.14 (0, 82.9) [0.38, 15.6]	1.3 (0, 8.1) [0, 7.9]
VE1_Dt	0.05 (0, 0.31) [0, 0.14]	0.07 (0, 0.16) [0.01, 0.11]	0 (0, 0.11) [0, 0.03]
roberta_embedding_mean	0.0001 (0, 0.0004) [0.0001, 0.0002]	0.0001 (0, 0.0003) [0.0001, 0.0002]	0.0002 (0, 0.0003) [0.0002, 0.0003]
BCUTp-1l	4.48 (2.12, 7.21) [3.96, 4.80]	4.16 (3.71, 5.63) [3.94, 4.60]	4.47 (3.88, 5.60) [4.41, 4.65]
HomoLumoGap:AM1:Cor3D:ori1	-9.65 (-15.1, 0) [-10.5, -9.06]	-10.1 (-14.2, -7.59) [-10.8, -9.13]	-10.7 (-11.5, -8.72) [-10.7, -9.72]
nO	2 (0, 16) [1, 2]	1 (0, 5) [1, 2.25]	3 (1, 11) [2, 8]
MDEC-12	1.37 (0, 13.5) [0, 2.84]	2.12 (0, 7.08) [0, 3.19]	0 (0, 5.23) [0, 0.96]
ALogp2	0.56 (0, 86.7) [0.11, 1.98]	1.69 (0, 16.0) [0.65, 2.60]	6.10 (0.50, 56.2) [0.85, 11.5]
BCUTc-1l	-0.35 (-0.42, -0.13) [-0.39, -0.30]	-0.33 (-0.42, 0.24) [-0.39, -0.30]	-0.39 (-0.41, -0.38) [-0.40, -0.39]
MLFER_S	1.05 (0.13, 3.75) [0.81, 1.42]	0.81 (0.41, 2.76) [0.69, 1.42]	1.61 (0.49, 2.71) [1.14, 2.36]
MDEC-11	0 (0, 3.14) [0, 0.25]	0.33 (0, 1.10) [0, 0.72]	0 (0, 1.28) [0, 0]

The values are presented as median (range) [interquartile range].

The features are sorted in order of SHAP values.

†The categorical variable 'SENS-IS_cat' has been excluded.

Table S3. Distribution of features included in the model (strong vs. weak sensitizer).

Feature†	Training set	Testing set (Correct prediction for strong and weak sensitizer)	Testing set (Incorrect prediction for strong and weak sensitizer)
	N = 65	N = 14	N = 3
C-peptide	36.8 (0, 100) [5.94, 96.4]	20.5 (0, 100) [5.88, 68.9]	31.9 (21.5, 52.3) [26.7, 42.1]
EC1.5	60.0 (0.5, 2000) [11.8, 424.4]	76.7 (1.8, 2000) [11.8, 1827.2]	45.4 (16.6, 60.5) [31.0, 53.0]
TSRW	9.15 (2.56, 11.0) [8.54, 9.49]	8.97 (8.34, 10.8) [8.72, 9.32]	9.15 (8.15, 10.3) [8.65, 9.75]
C1SP3	1 (0, 6) [0, 2]	2.5 (0, 5) [1, 3]	2 (1, 3) [1.5, 2.5]
VE1_Dt	0.05 (0, 0.28) [0, 0.10]	0.06 (0, 0.16) [0.01, 0.11]	0 (0, 0.12) [0, 0.06]
BP	251.6 (-19.1, 488.1) [205.3, 307.1]	229.0 (80.7, 565.3) [200.9, 262.8]	240.0 (170.7, 932.2) [205.4, 586.1]
RotBFrac	0.27 (0, 0.67) [0.15, 0.40]	0.19 (0, 0.54) [0.09, 0.40]	0.25 (0.18, 0.36) [0.22, 0.30]
SIC0	0.30 (0.21, 0.75) [0.25, 0.36]	0.28 (0.20, 0.44) [0.25, 0.32]	0.27 (0.25, 0.36) [0.26, 0.32]
MDEC-22	4.72 (0, 18.6) [1.65, 9.13]	3.22 (0.36, 11.5) [1.65, 6.93]	2.38 (0.40, 4.88) [1.39, 3.63]
K-peptide	8.5 (0, 93.7) [0, 20.4]	8.0 (0, 42.4) [0.2, 15.1]	3.1 (1.3, 13.8) [2.2, 8.4]

The values are presented as median (range) [interquartile range].

The features are sorted in order of SHAP values.

Figure S1. The feature importance of a classifier distinguishing between sensitizer and non-sensitizer

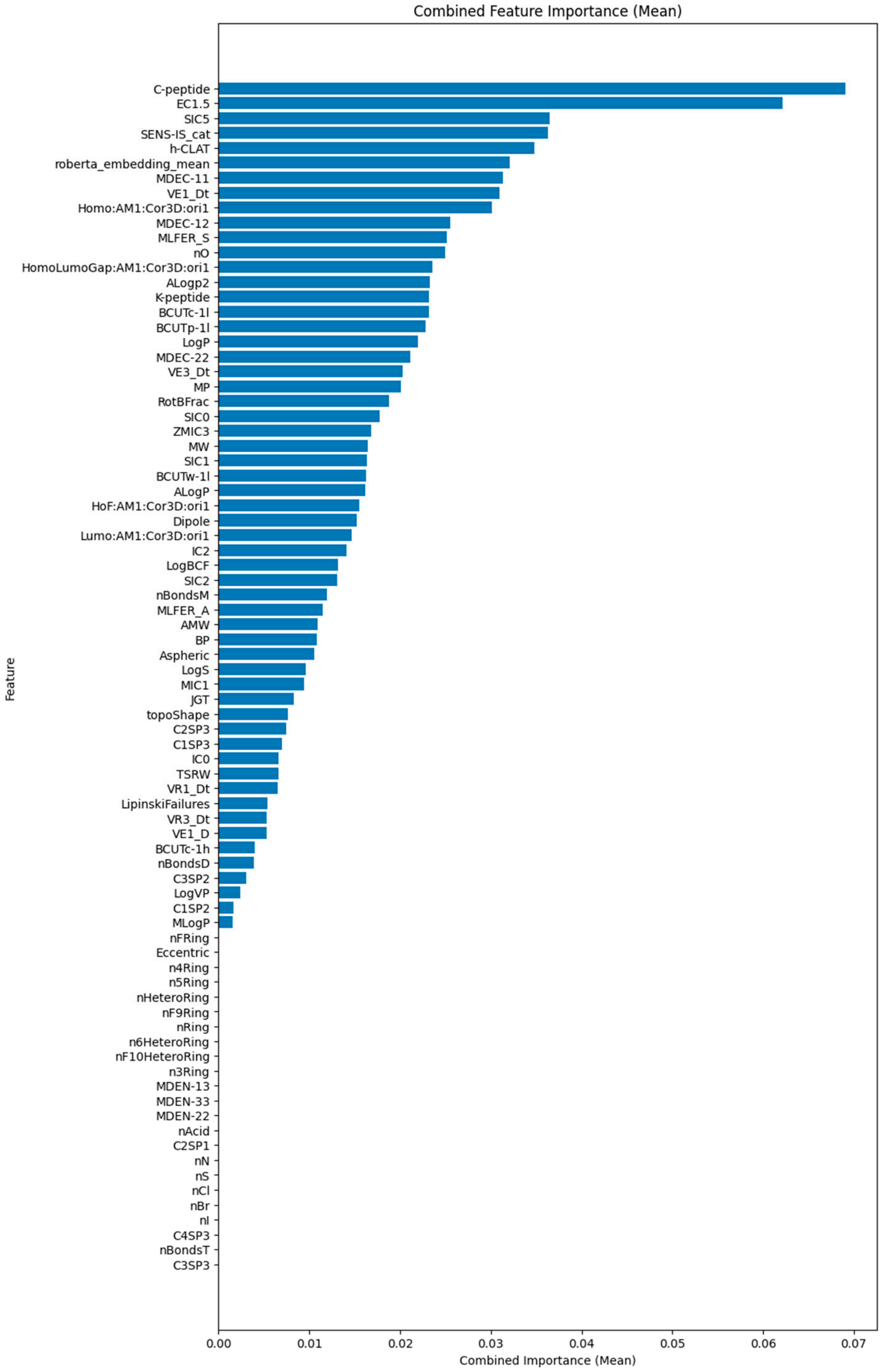

Figure S2. The feature importance of a classifier distinguishing between strong and weak sensitizer

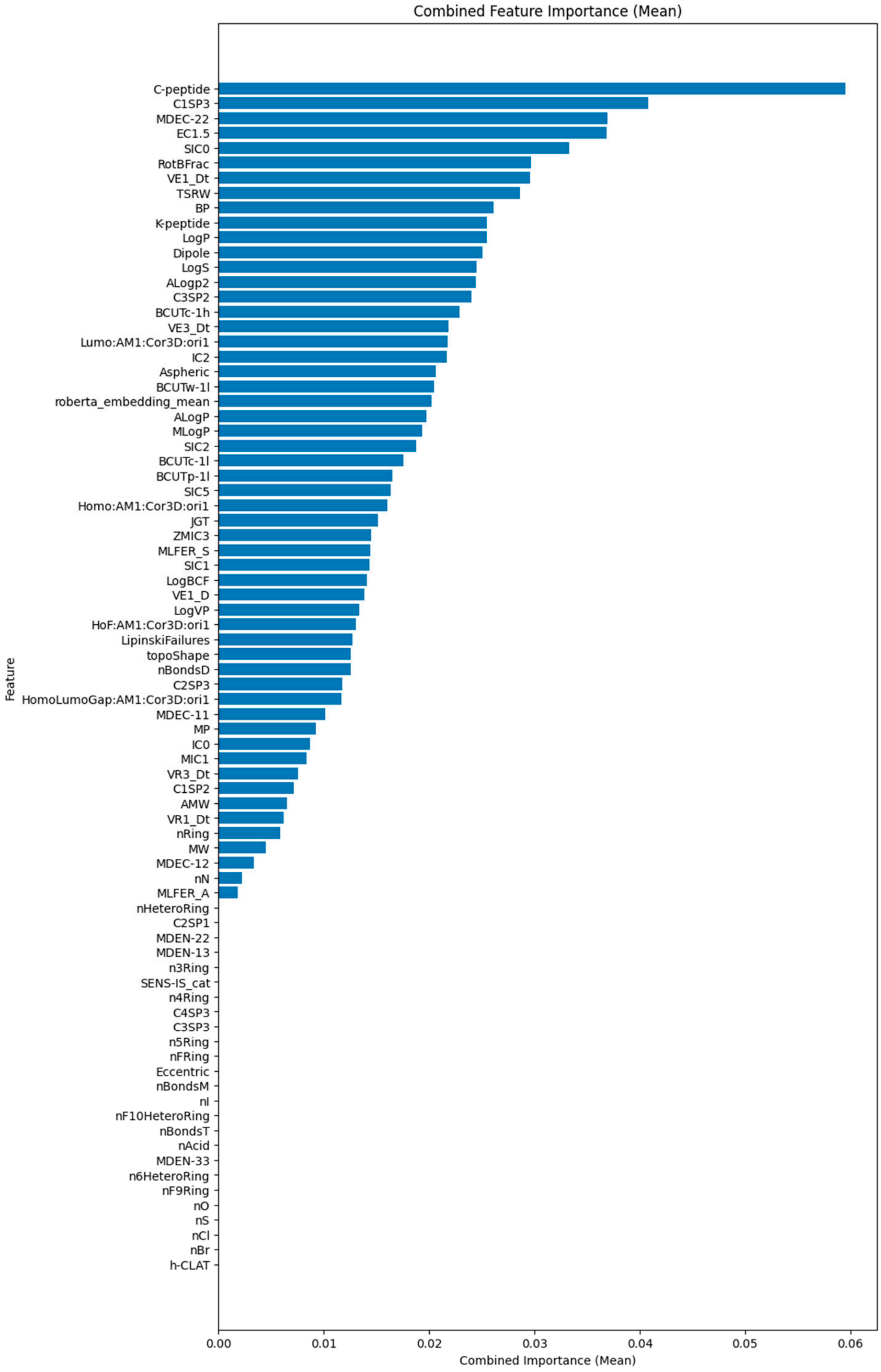

Figure S3. The average SHAP values for a classifier distinguishing between sensitizer and non-sensitizer.

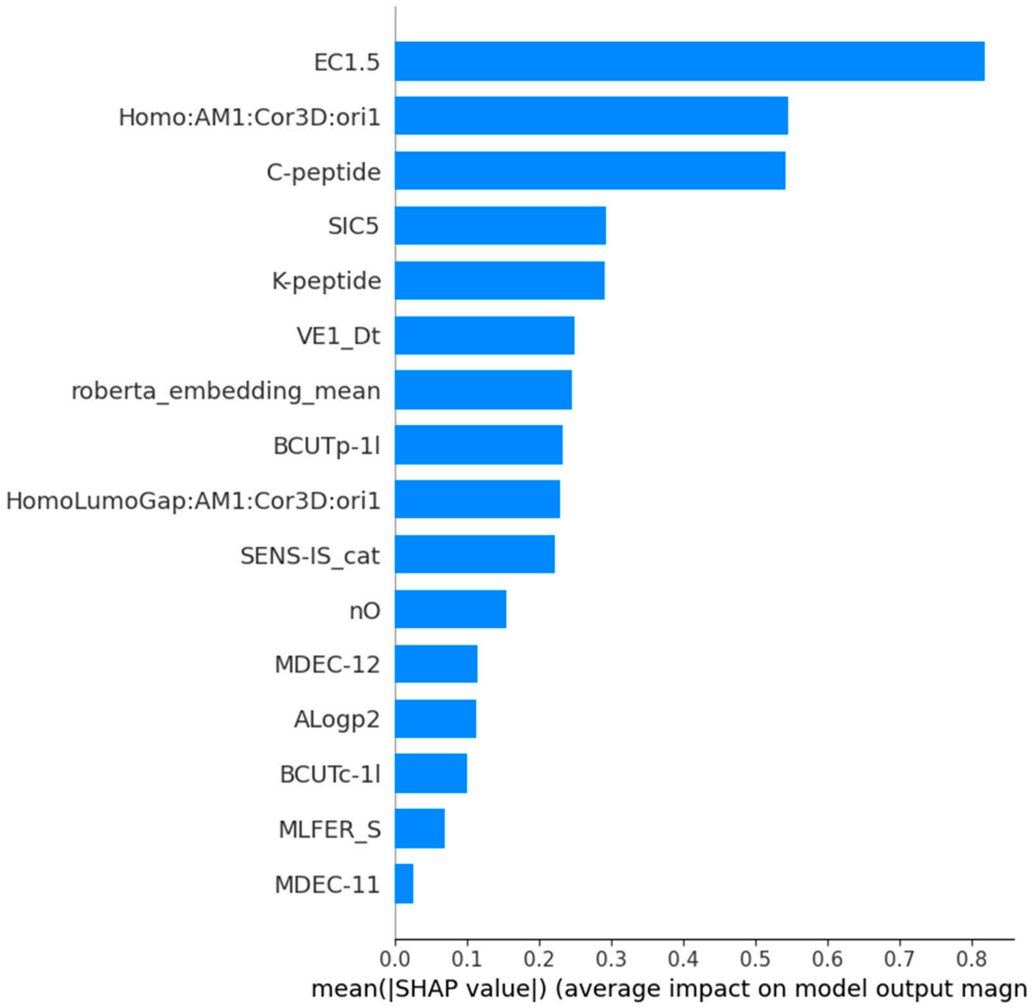

Figure S4. The average SHAP values for a classifier distinguishing between strong and weak sensitizer.

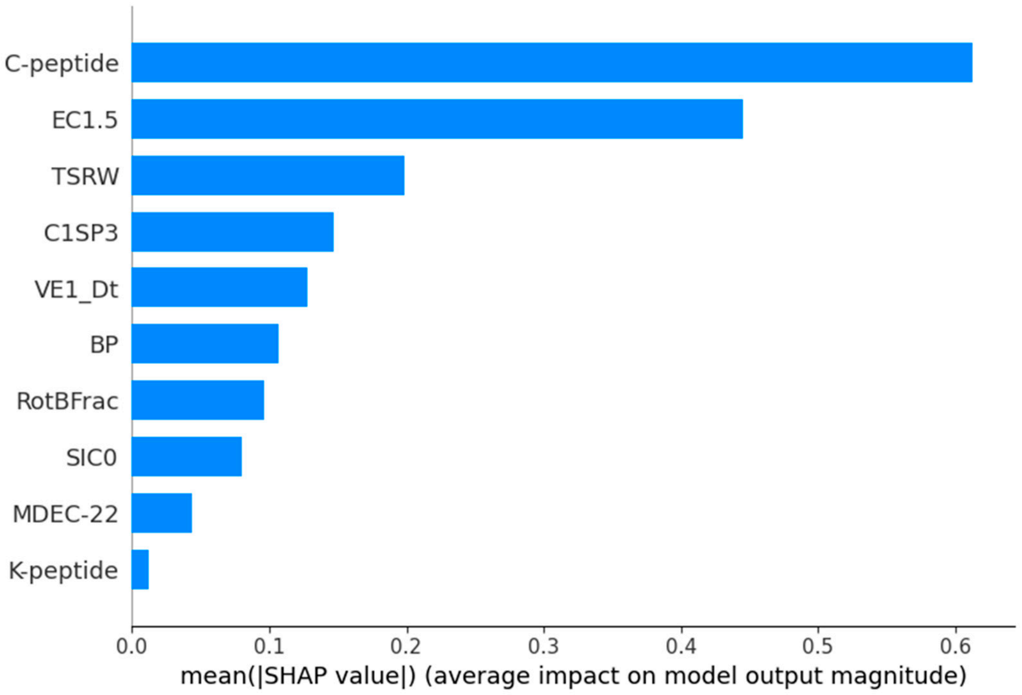

Figure S5. SHAP force plot for test substances and predicted results (sensitizer vs. non-sensitizer).

(1) Vanillin (non-sensitizer): false positive

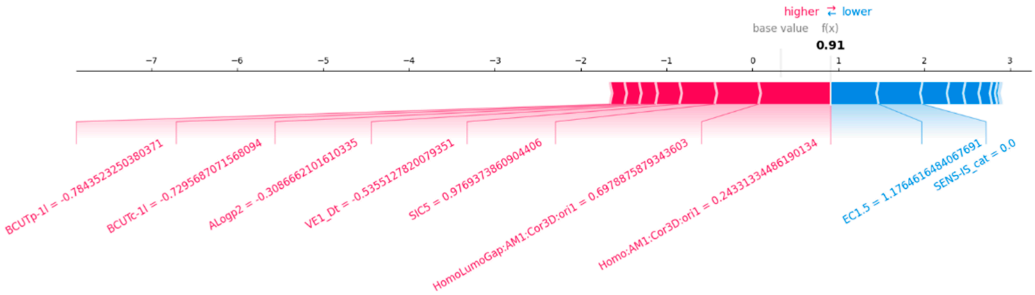

(2) Carvone (sensitizer): true positive

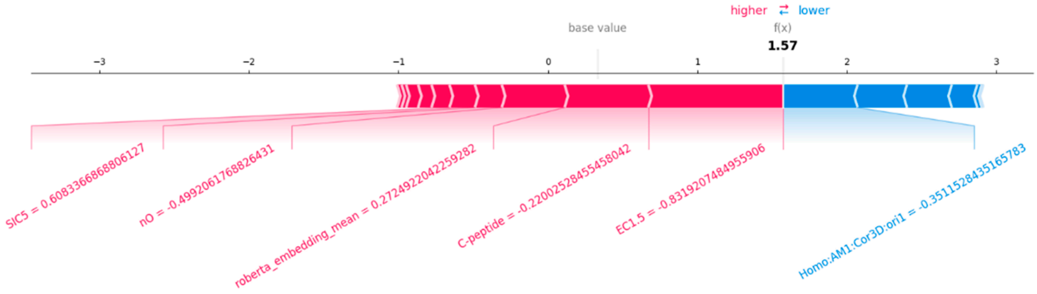

(3) Hydrocortisone (non-sensitizer): true negative

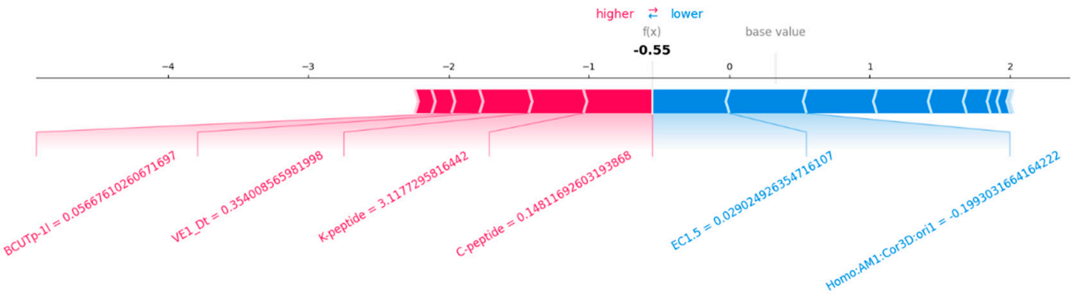

(4) p-Mentha-1,8-dien-7-al (sensitizer): true positive

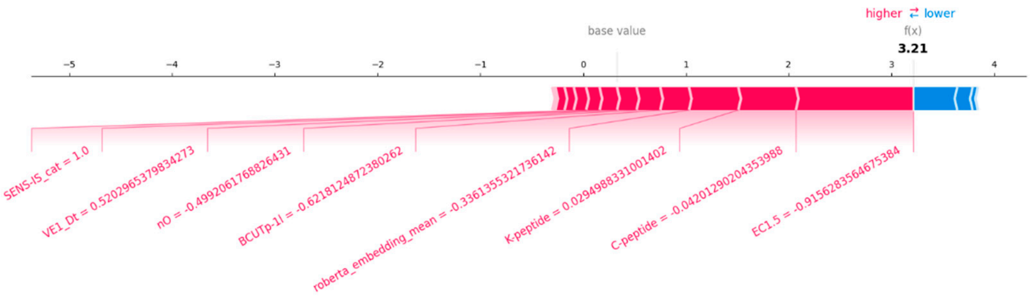

(5) Imidazolidinyl urea (sensitizer): false negative

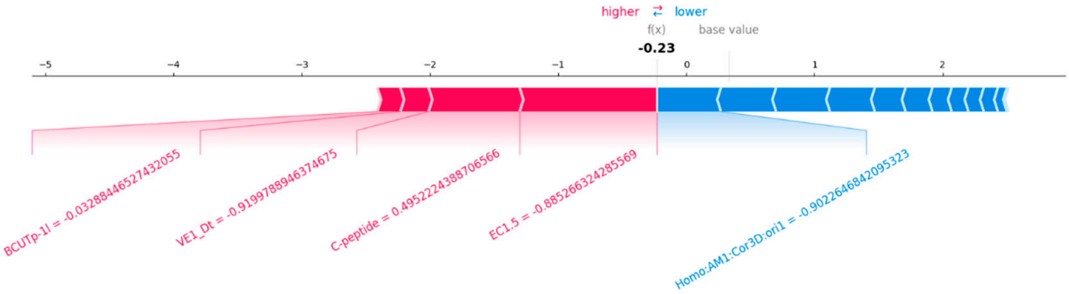

(6) Sodium lauryl sulfate (non-sensitizer): true negative

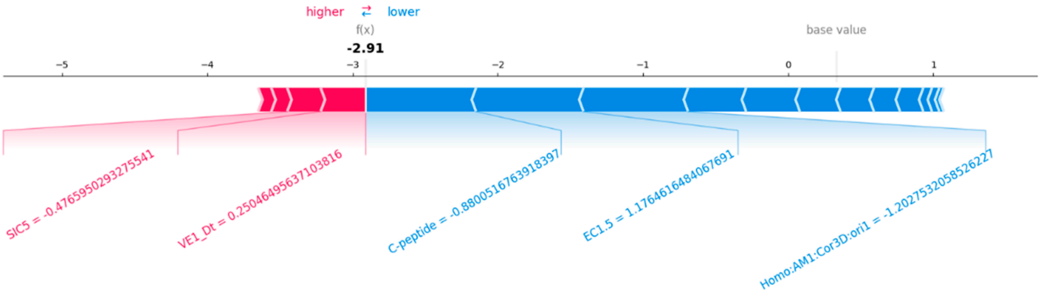

(7) Farnesol (sensitizer): true positive

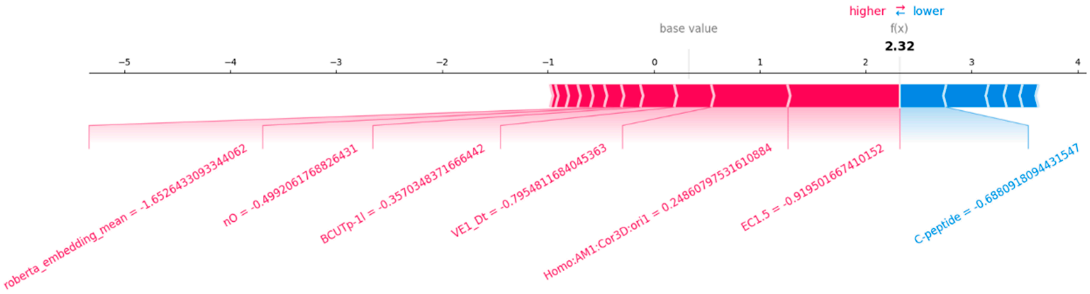

(8) Isopropanol (non-sensitizer): true negative

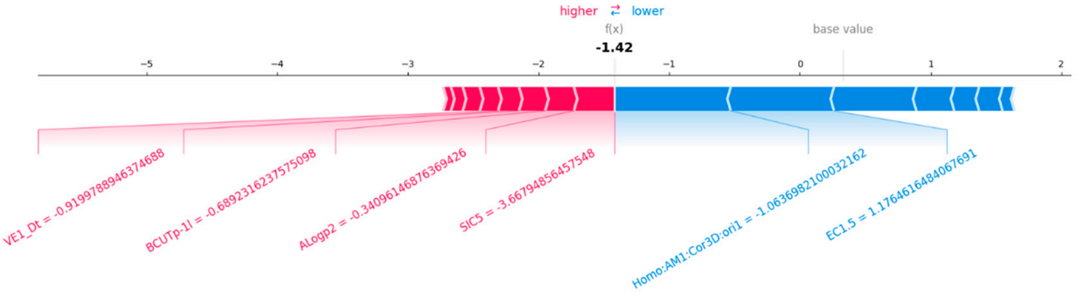

(9) 6-Methyl-3,5-heptadien-2-one (sensitizer): true positive

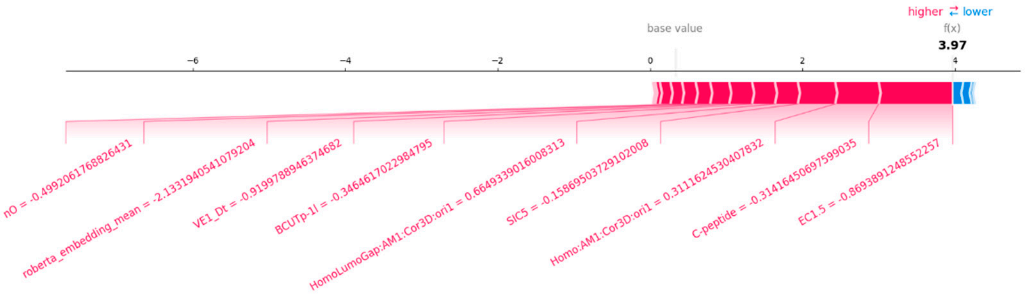

(10) Kanamycin sulfate (sensitizer): false negative

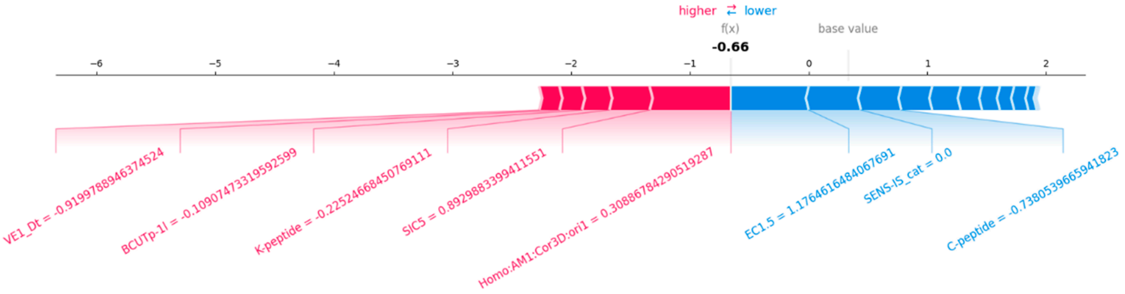

(11) Ethyleneglycol dimethacrylate (sensitizer): true positive

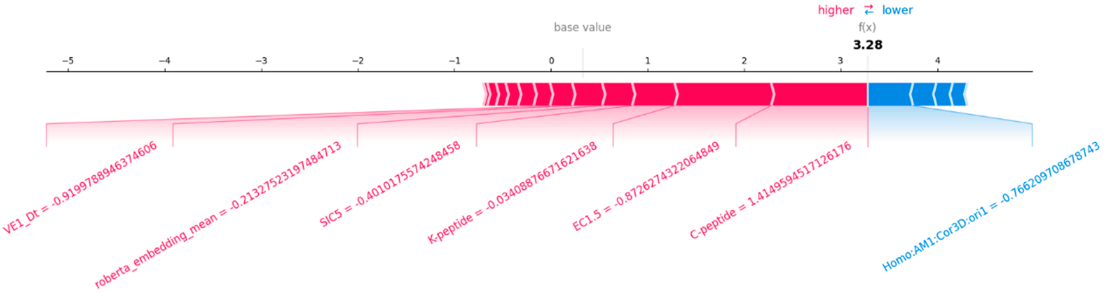

(12) Penicillin G (sensitizer): true positive

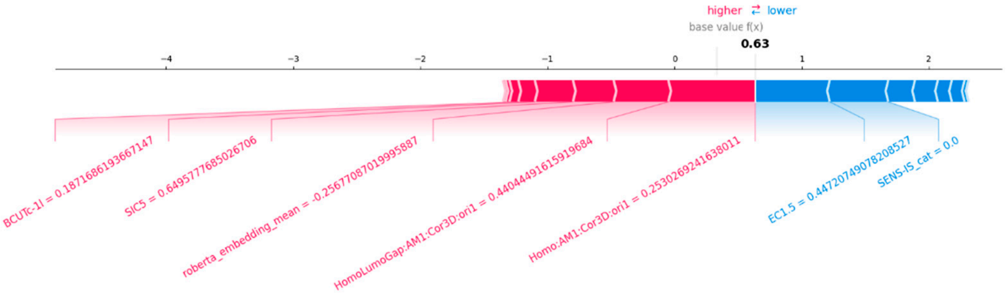

(13) 1,4-Phenylenediamine (sensitizer): true positive

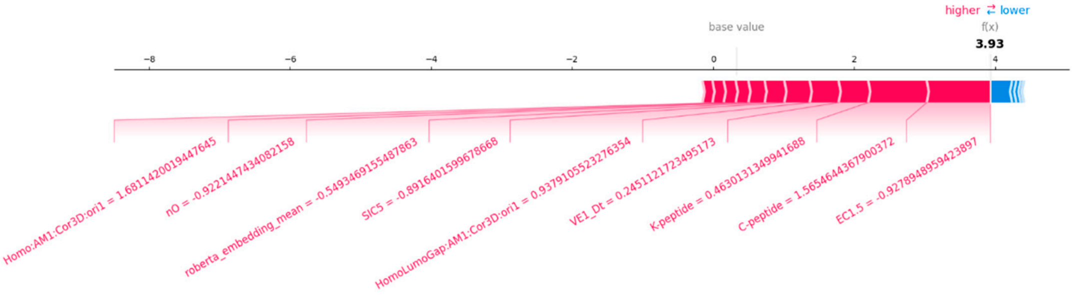

(14) Citronellol (non-sensitizer): true negative

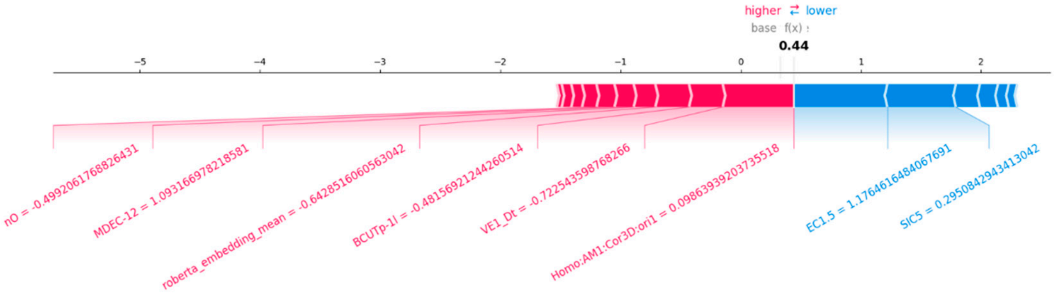

(15) Isocyclocitral (sensitizer): true positive

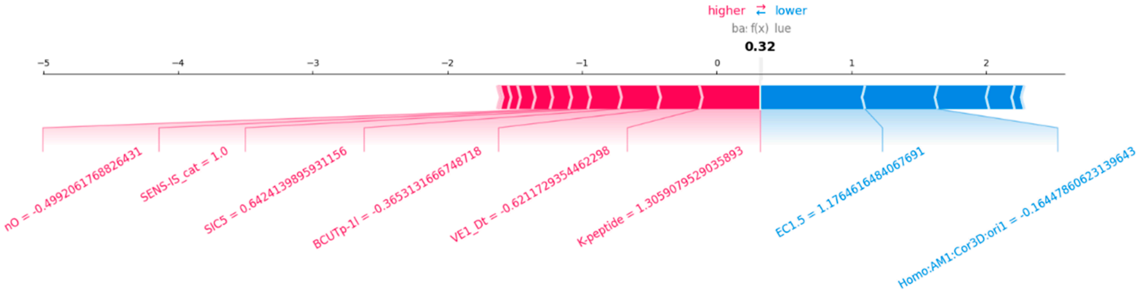

(16) Diethanolamine (non-sensitizer): true negative

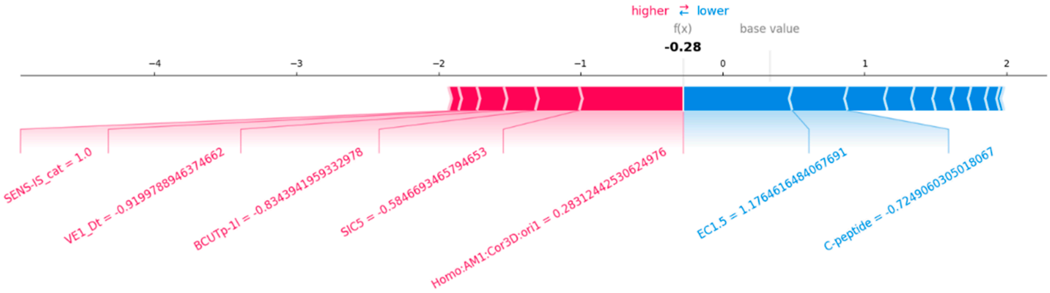

(17) Toluene diamine sulphate (sensitizer): true positive

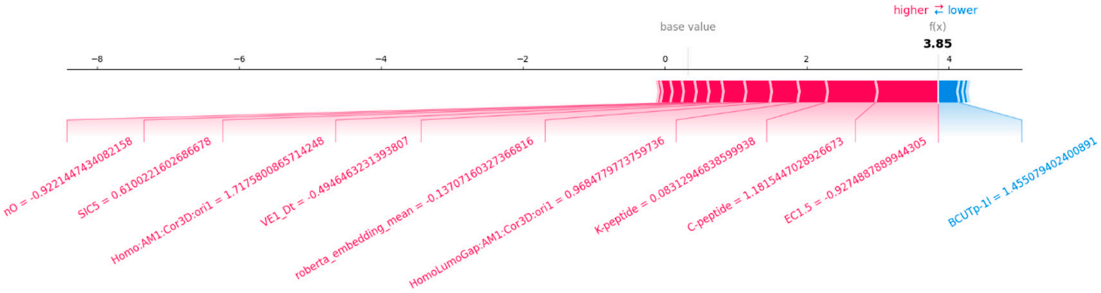

(18) Methyl 2-nonynoate (sensitizer): true positive

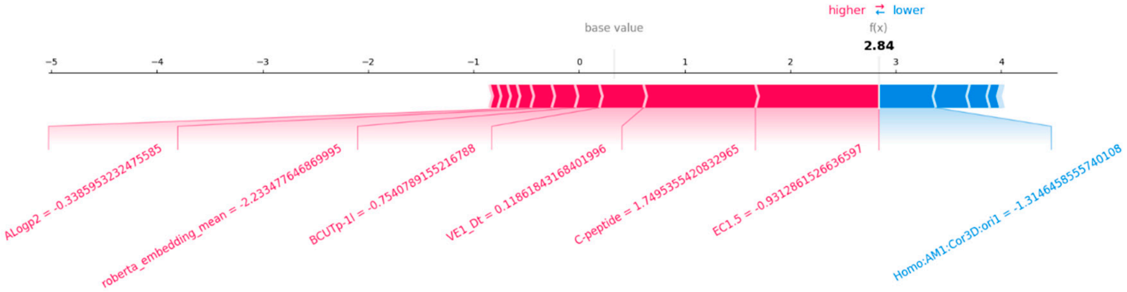

(19) Salicylic acid (non-sensitizer): true negative

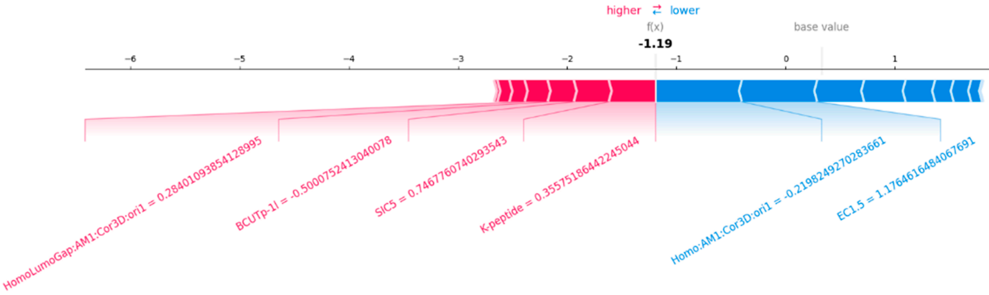

(20) Resorcinol (sensitizer): false negative

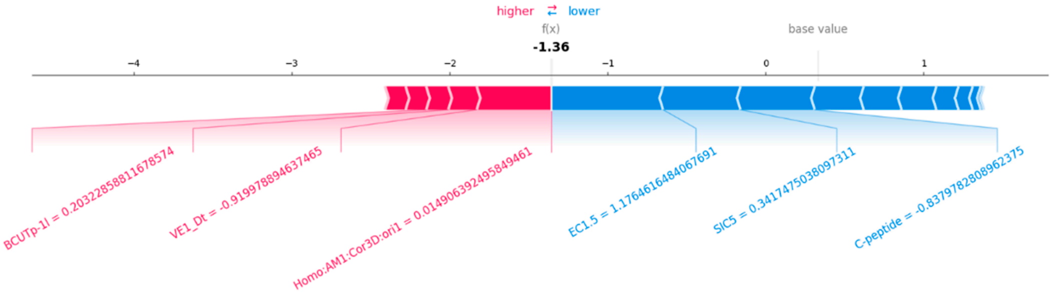

(21) Citral (sensitizer): true positive

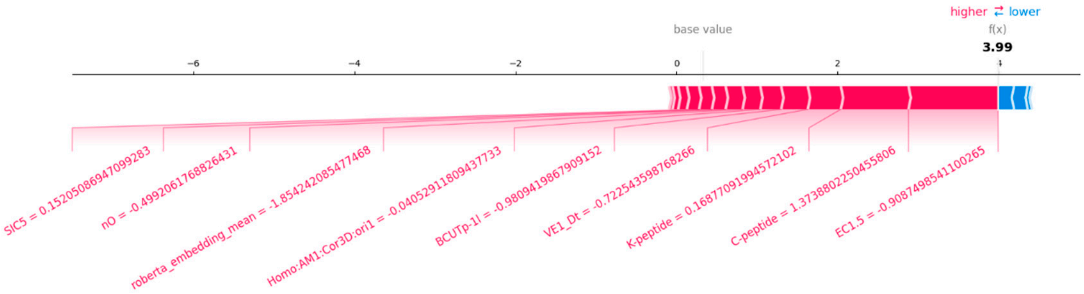

(22) 2-Nitro-1,4-phenylenediamine (sensitizer): true positive

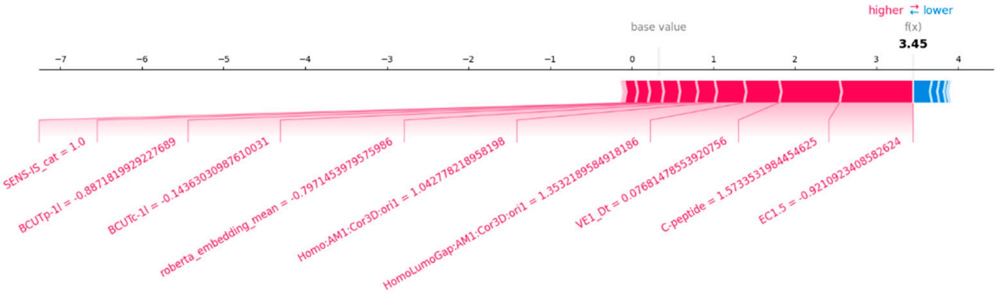

(23) Octanoic acid (non-sensitizer): true negative

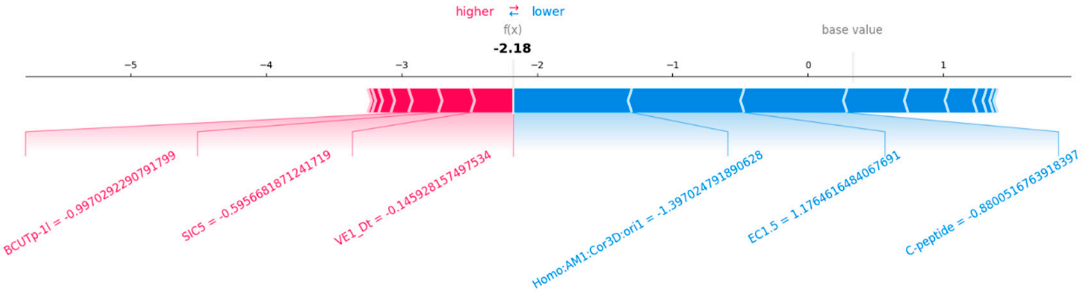

(24) Cinnamic alcohol (sensitizer): true positive

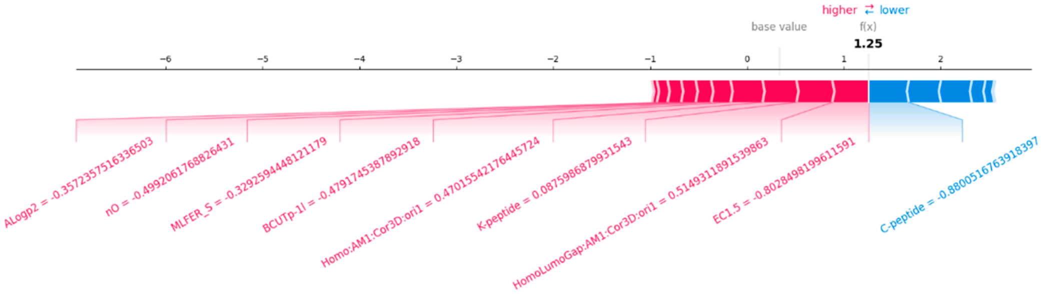

(25) Linalool (sensitizer): false negative

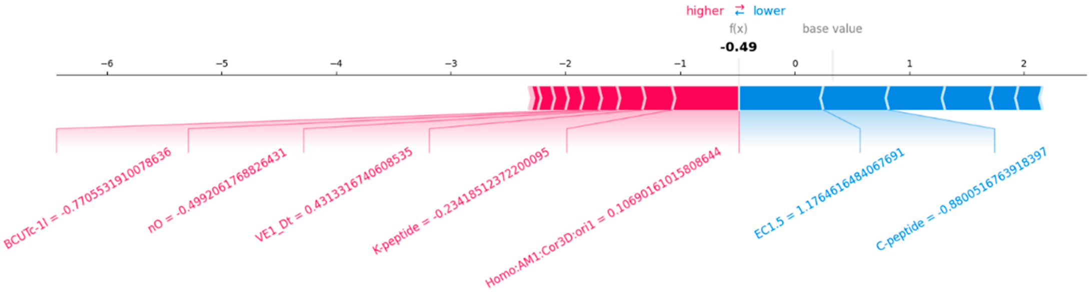

Figure S6. SHAP force plot for test substances and predicted results (strong vs. weak sensitizer).

(1) Carvone (weak sensitizer): true negative

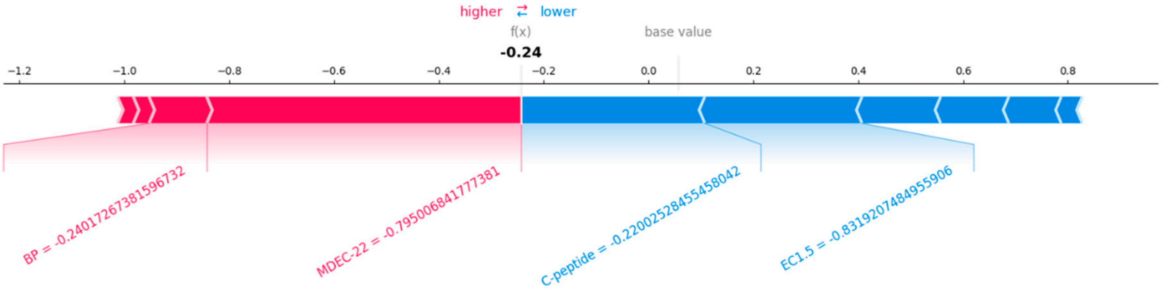

(2) p-Mentha-1,8-dien-7-al (weak sensitizer): false positive

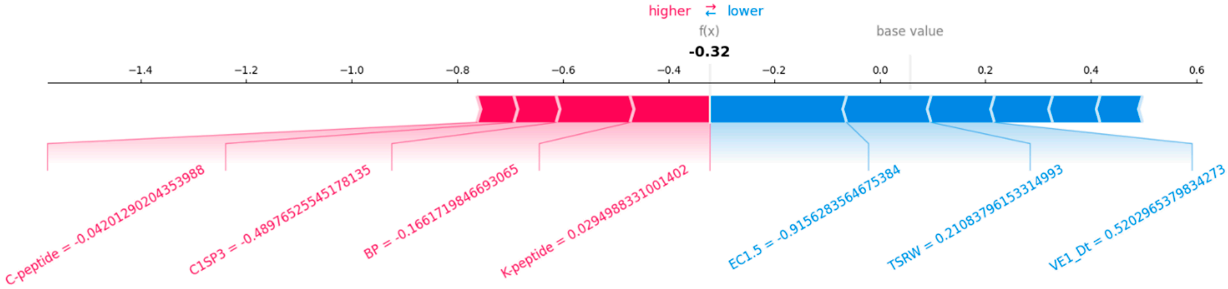

(3) Imidazolidinyl urea (weak sensitizer): false positive

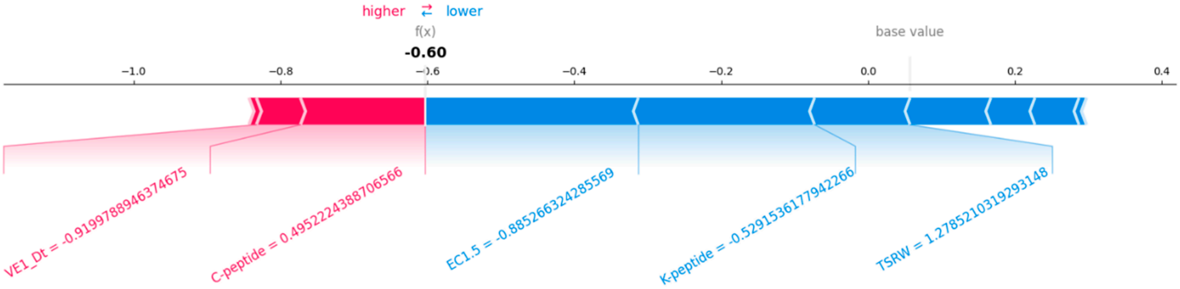

(4) Farnesol (weak sensitizer): true negative

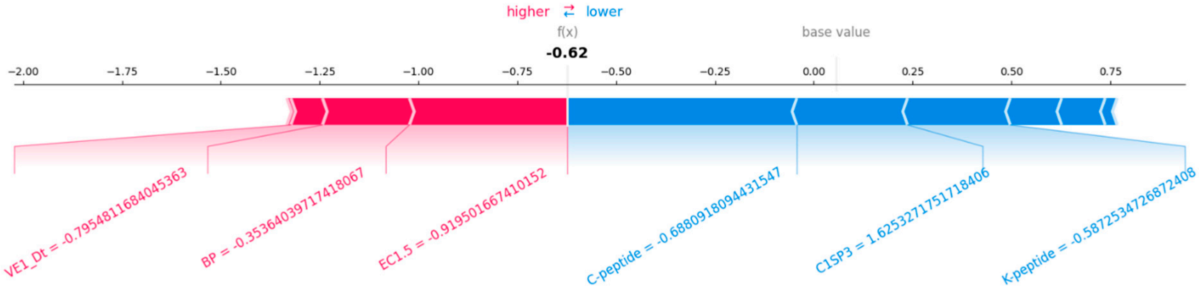

(5) 6-Methyl-3,5-heptadien-2-one (strong sensitizer): false negative

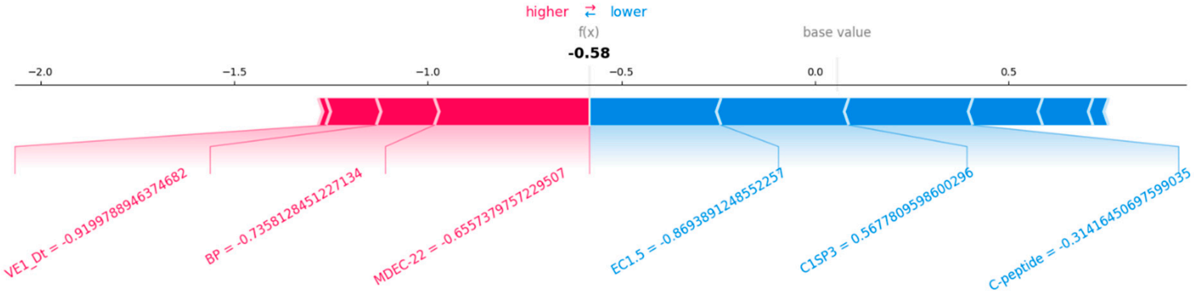

(6) Kanamycin sulfate (weak sensitizer): true negative

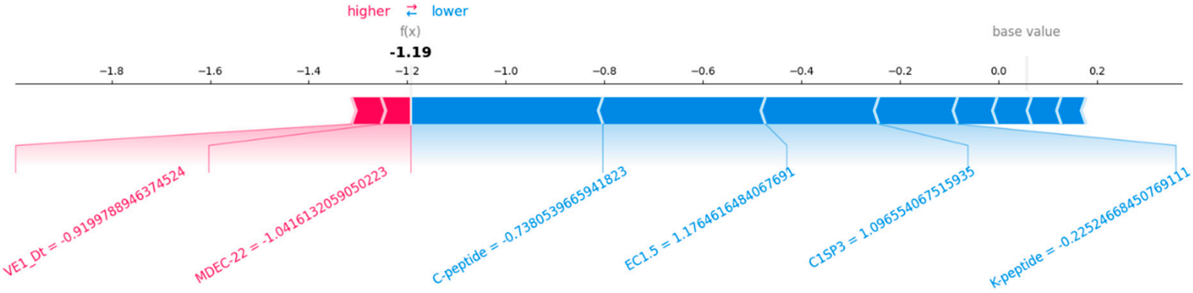

(7) Ethyleneglycol dimethacrylate (weak sensitizer): true negative

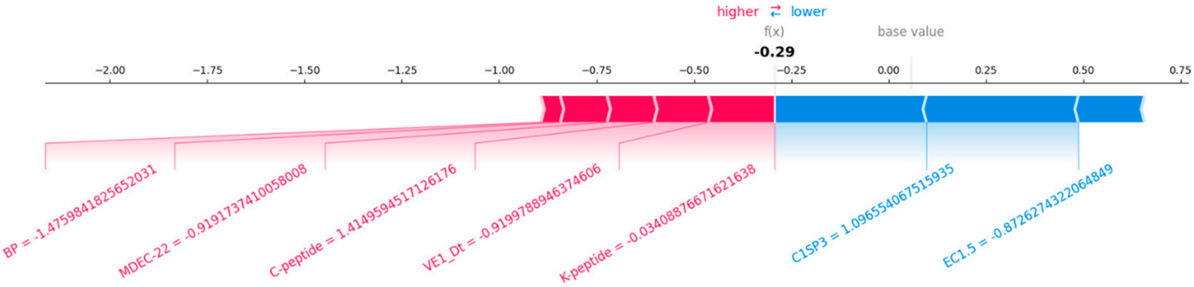

(8) Penicillin G (weak sensitizer): true negative

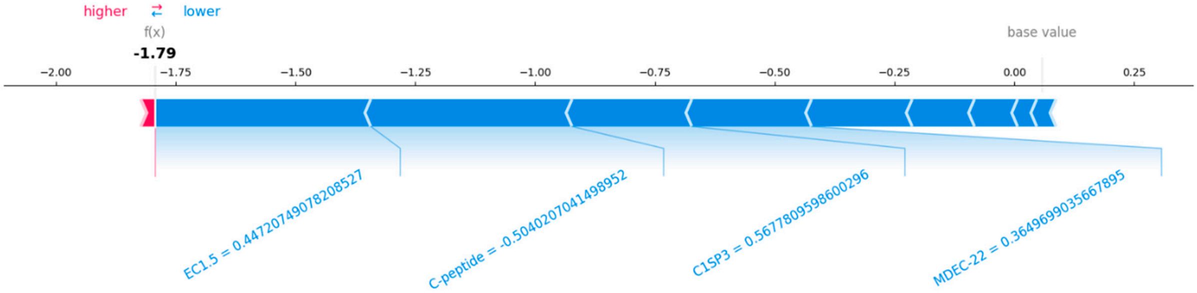

(9) 1,4-Phenylenediamine (strong sensitizer): true positive

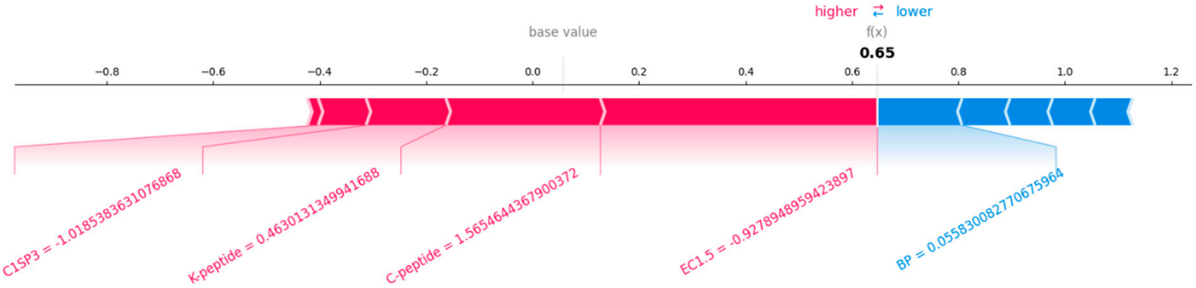

(10) Isocyclocitral (weak sensitizer): true negative

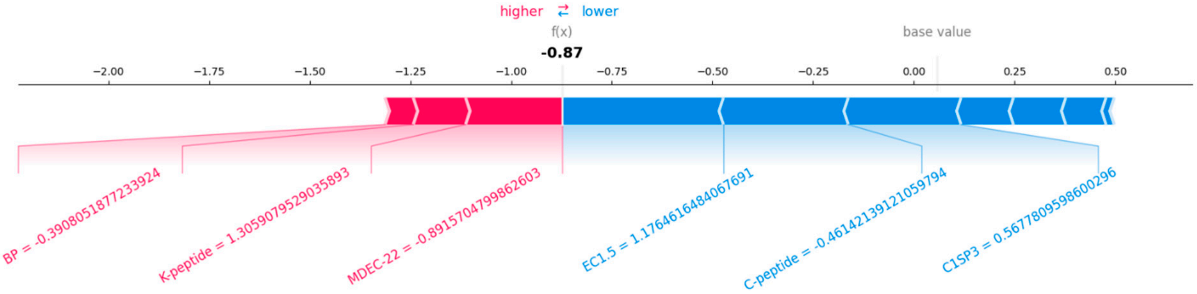

(11) Toluene diamine sulphate (strong sensitizer): true positive

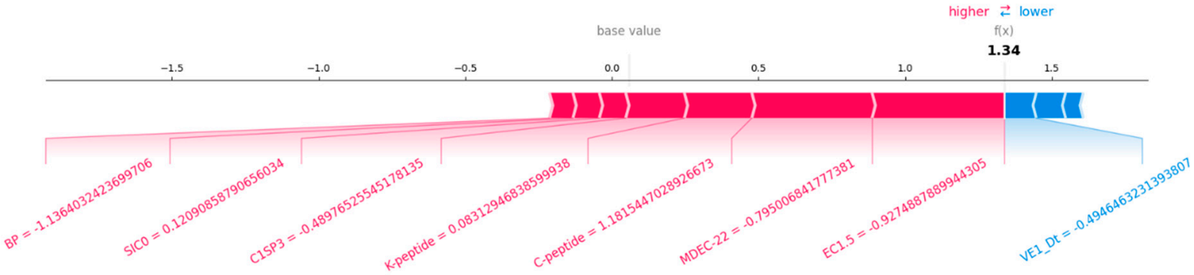

(12) Methyl 2-nonynoate (strong sensitizer): true positive

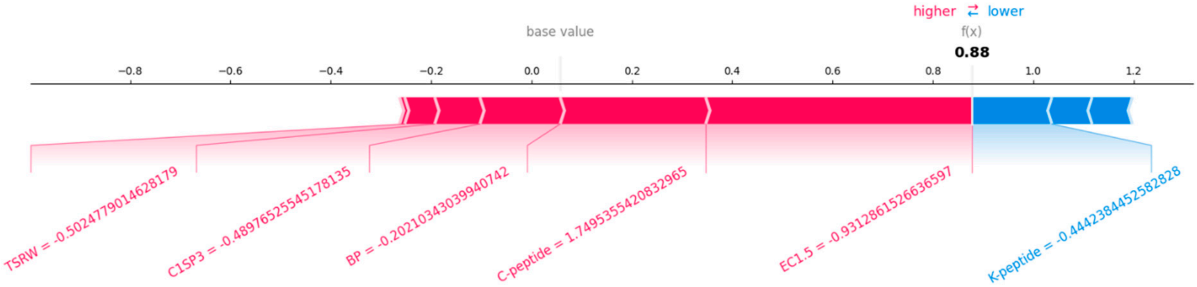

(13) Resorcinol (weak sensitizer): true negative

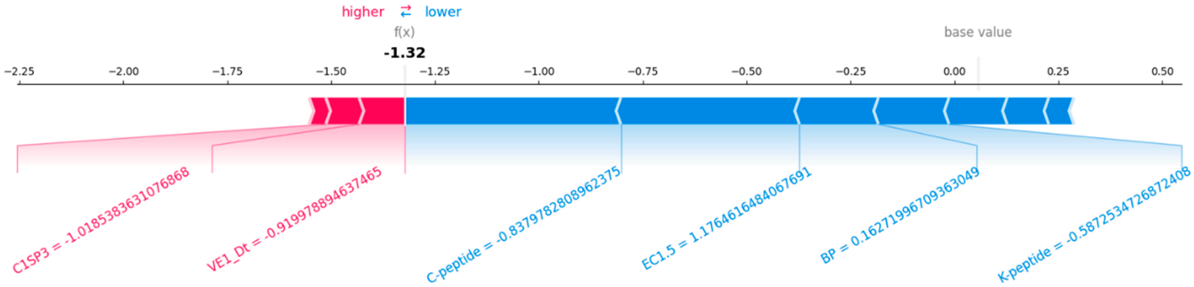

(14) Citral (weak sensitizer): true negative

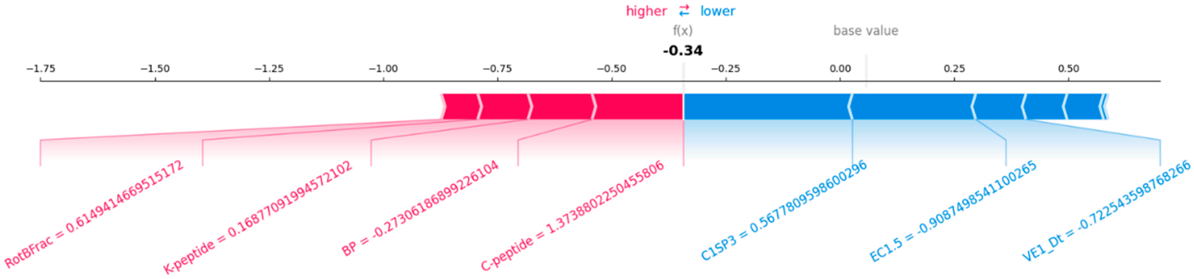

(15) 2-Nitro-1,4-phenylenediamine (strong sensitizer): true positive

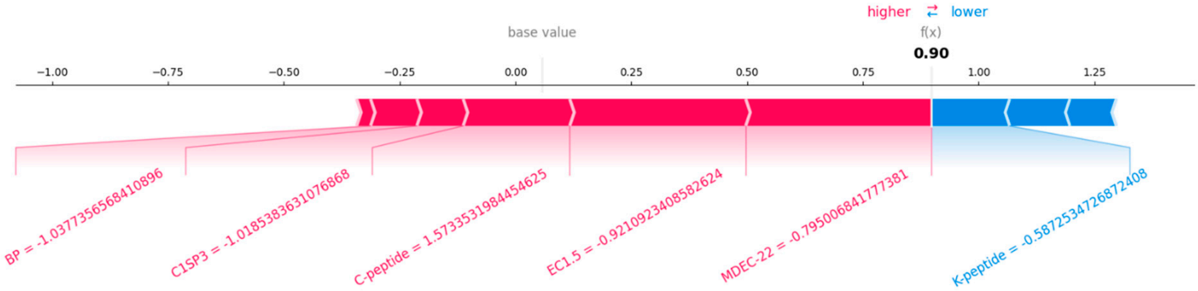

(16) Cinnamic alcohol (weak sensitizer): true negative

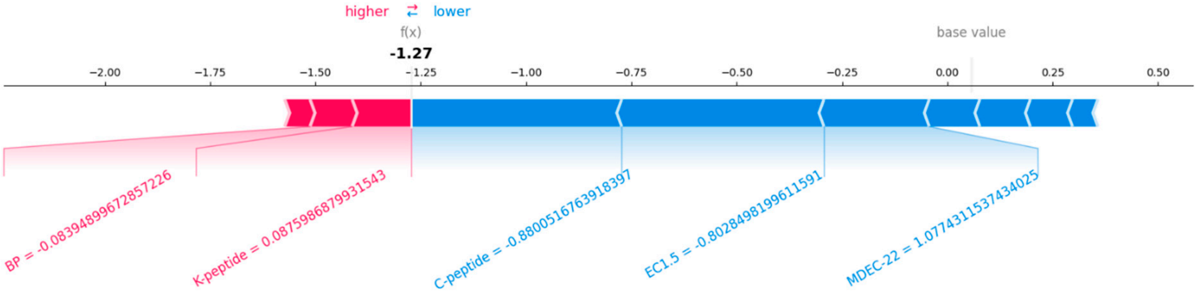

(17) Linalool (weak sensitizer): true negative

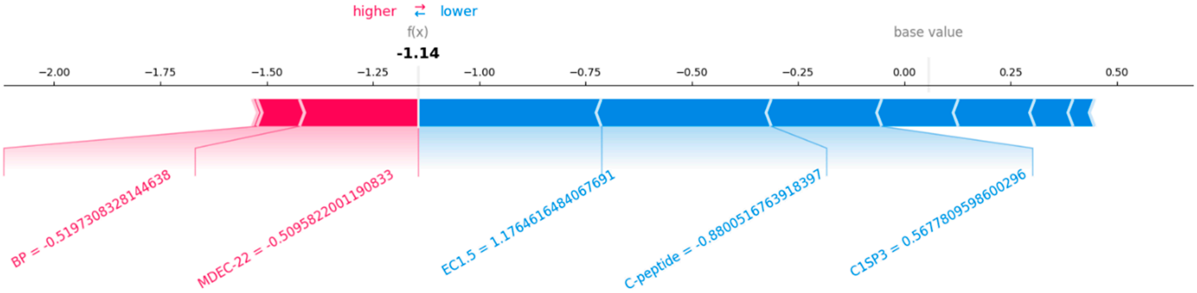

Supplement: Supplementary file 1 [file toxics-12-00153-s001.zip › Supplementary Materials.pdf]
